# Probiotics Protect Mice from Ovariectomy-Induced Cortical Bone Loss

**DOI:** 10.1371/journal.pone.0092368

**Published:** 2014-03-17

**Authors:** Claes Ohlsson, Cecilia Engdahl, Frida Fåk, Annica Andersson, Sara H. Windahl, Helen H. Farman, Sofia Movérare-Skrtic, Ulrika Islander, Klara Sjögren

**Affiliations:** 1 Centre for Bone and Arthritis Research, Institute of Medicine, Sahlgrenska Academy at University of Gothenburg, Gothenburg, Sweden; 2 Department of Rheumatology and Inflammation Research, Institute of Medicine, Sahlgrenska Academy at University of Gothenburg, Gothenburg, Sweden; 3 Applied Nutrition and Food Chemistry, Department of Food Technology, Engineering and Nutrition, Lund University, Lund, Sweden; French National Centre for Scientific Research, France

## Abstract

The gut microbiota (GM) modulates the hosts metabolism and immune system. Probiotic bacteria are defined as live microorganisms which when administered in adequate amounts confer a health benefit on the host and can alter the composition of the GM. Germ-free mice have increased bone mass associated with reduced bone resorption indicating that the GM also regulates bone mass. Ovariectomy (ovx) results in bone loss associated with altered immune status. The purpose of this study was to determine if probiotic treatment protects mice from ovx-induced bone loss. Mice were treated with either a single *Lactobacillus* (L) strain, *L. paracasei* DSM13434 (L. para) or a mixture of three strains, *L. paracasei* DSM13434, *L. plantarum* DSM 15312 and DSM 15313 (L. mix) given in the drinking water during 6 weeks, starting two weeks before ovx. Both the L. para and the L. mix treatment protected mice from ovx-induced cortical bone loss and bone resorption. Cortical bone mineral content was higher in both L. para and L. mix treated ovx mice compared to vehicle (veh) treated ovx mice. Serum levels of the resorption marker C-terminal telopeptides and the urinary fractional excretion of calcium were increased by ovx in the veh treated but not in the L. para or the L. mix treated mice. Probiotic treatment reduced the expression of the two inflammatory cytokines, TNFα and IL-1β, and increased the expression of OPG, a potent inhibitor of osteoclastogenesis, in cortical bone of ovx mice. In addition, ovx decreased the frequency of regulatory T cells in bone marrow of veh treated but not probiotic treated mice. In conclusion, treatment with L. para or the L. mix prevents ovx-induced cortical bone loss. Our findings indicate that these probiotic treatments alter the immune status in bone resulting in attenuated bone resorption in ovx mice.

## Introduction

Fractures caused by osteoporosis constitute a major health concern and result in a huge economic burden on health care systems. In Sweden, the lifetime risk of any osteoporotic fracture is 47% and 24% in women and men, respectively [1]. In USA, the risk has been reported to be 40% and 13% in white women and men, respectively and fractures are associated with significant mortality and morbidity [2]. Cortical bone is the major contributor to non-vertebral fracture risk and comprises more than 80% of the skeleton.

The skeleton is remodeled by bone forming osteoblasts (OBs) and bone resorbing osteoclasts (OCLs). Macrophage colony stimulating factor (M-CSF) increases proliferation and survival of OCLs precursor cells as well as up-regulates expression of receptor activator of nuclear factor-κB (RANK) in OCL. This allows RANK ligand (RANKL) to bind and start the signaling cascade that leads to OCL formation. The effect of RANKL can be inhibited by Osteoprotegerin (OPG), which is a decoy receptor for RANKL [3].

The association between inflammation and bone loss is well established. In autoimmune diseases, osteoclastic bone resorption is driven by inflammatory cytokines produced by immune cells e.g. activated T cells [4]. In addition, low-grade systemic inflammation, indicated by moderately elevated serum levels of high sensitivity C-reactive protein (hsCRP), associates with low bone mineral density (BMD), elevated bone resorption and increased fracture risk [5]–[8]. The estrogen deficiency that occurs after menopause results in increased formation and prolonged survival of OCLs. This is suggested to be due to a number of factors including loss of the immunosuppressive effects of estrogen, resulting in increased production of cytokines promoting osteoclastogenesis, and direct effects of estrogen on OCLs [9], [10]. In line with these data, blockade of the inflammatory cytokines TNFα and IL-1 leads to a decrease in bone resorption markers in early postmenopausal women [11].

In recent years, the importance of the gut microbiota (GM) for both health and disease has been intensively studied. The GM constitutes of trillions of bacteria which collectively contain 150-fold more genes than our human genome. It is acquired at birth and, although a distinct entity, it has clearly coevolved with the human genome and can be considered a multicellular organ that communicates with and affects its host in numerous ways [12]. The composition of the GM is modulated by a number of environmental factors such as diet and antibiotic treatments. Molecules produced by the gut bacteria can be both beneficial and harmful and are known to affect the host's immune system [13]. Perturbed microbial composition has been postulated to be involved in a range of inflammatory conditions, within and outside the gut including Crohn's disease, ulcerative colitis, rheumatoid arthritis, multiple sclerosis, diabetes, food allergies, eczema and asthma as well as obesity and the metabolic syndrome [13], [14]. We recently showed that absence of GM in germ-free (GF) mice leads to increased bone mass associated with reduced bone resorption and altered immune status in bone. Colonisation of GF mice with a normal gut microbiota led to a normalisation of bone mass and immune status in bone marrow [15]. A role of the GM for bone mass is supported by a recent study demonstrating that subtherapeutic antibiotic treatment in early life increases bone mass in young mice [16]. Although the low dose of antibiotics in this study did not cause a significant alteration in bacterial count it caused shifts in the composition of the GM. Furthermore, tetracycline treatment has been shown to prevent bone loss and improve mechanical properties of bone after ovariectomy (ovx) [17], [18]. These studies, demonstrate that antibiotic treatment has the capacity to influence both the GM composition and bone mass, supporting the notion that the GM is a regulator of bone homeostasis.

Probiotic bacteria are defined as live microorganisms which when administered in adequate amounts confer a health benefit on the host. Probiotics act by altering the composition or the metabolic activity of the GM [19]. The suggested underlying mechanisms for how probiotics contribute to health are manifold including increased solubility and absorption of minerals, enhanced barrier function and modulation of the immune system [20], [21]. Ovx in mice results in bone loss associated with altered immune status, resembling post-menopausal osteoporosis. The purpose of the present study was to determine if probiotic treatment protects mice from ovx-induced bone loss.

## Materials and Methods

### Ovx Mouse-model and Probiotic Treatment

**Figure 1:**
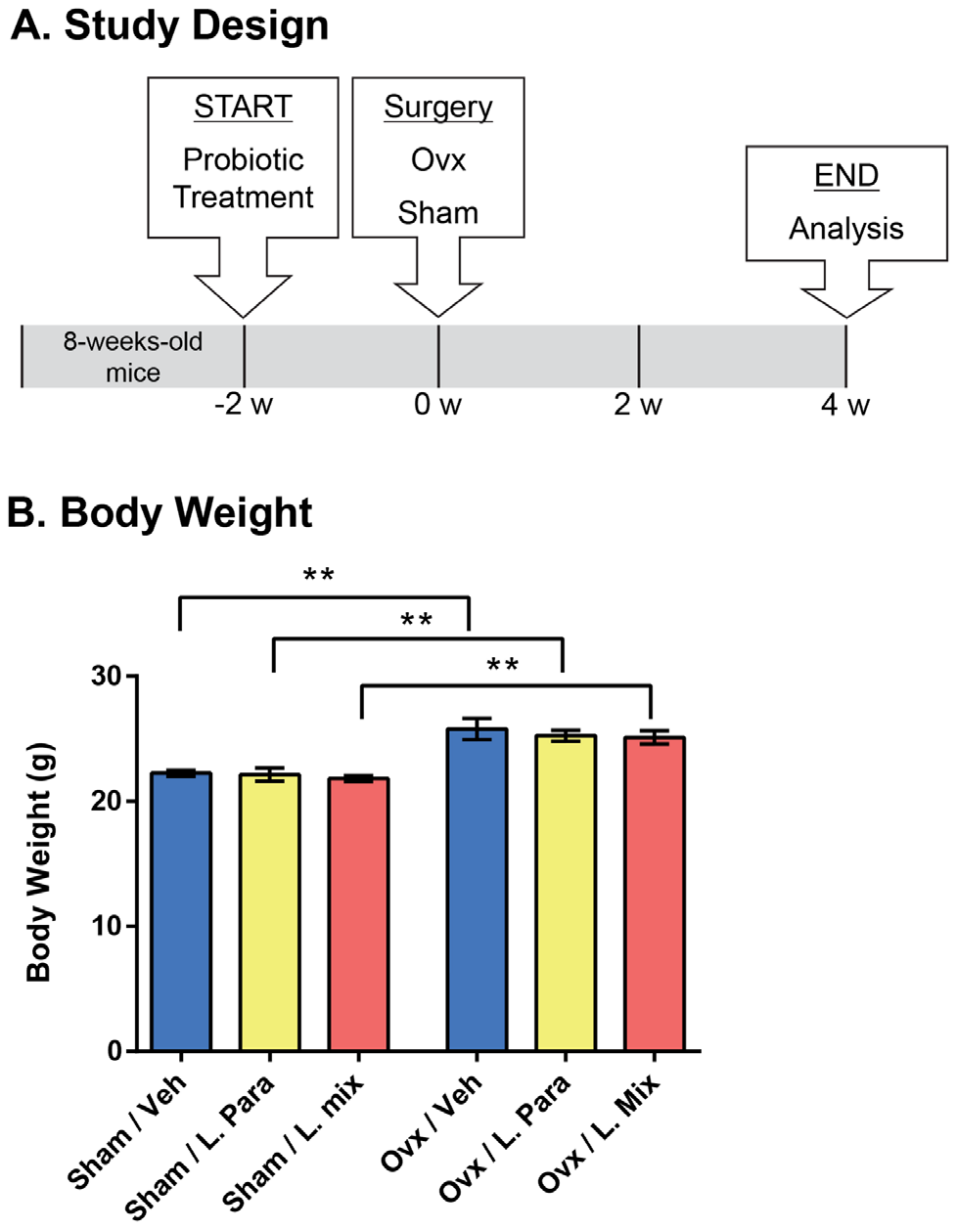
Study Design and Body Weight. Outline of study design (A). Eight-week-old mice were treated with either vehicle (veh), a single *Lactobacillus* (L) strain (L. para) or a mixture of three strains (L. mix) during 6 weeks, starting two weeks before ovx or sham surgery. The L. strains were given in the drinking water at a concentration of 10^9^ colony-forming units (cfu)/ml while control mice received tap water with vehicle. Mice were 14-week-old at the end of the study, when tissues were collected for later analysis. Ovx resulted in an expected increased body weight compared to sham mice that was not different after probiotic treatment (B). Results are given as mean±SEM (n = 9–10), ** p≤0.01. Students *t* test ovx vs. sham.

Six-week-old C57BL/6N female mice were purchased from Charles River (Germany). The mice were housed in a standard animal facility under controlled temperature (22°C) and photoperiod (12-h light, 12-h dark) and had free access to fresh water and soy-free food pellets R70 (Lactamin AB, Stockholm, Sweden). The ovx model for osteoporosis is included in the FDA guidelines for preclinical and clinical evaluation for agents used for the treatment of postmenopausal osteoporosis [22]. Probiotic treatment started two weeks before ovx to study the preventive effect of probiotic treatment on ovx induced bone-loss (Fig. 1A). Mice were treated with either a single *Lactobacillus* (L) strain, *L. paracasei* DSM13434 (L. para) or a mixture of three strains, *L. paracasei* DSM13434, *L. plantarum* DSM 15312 and DSM 15313 referred to as L. mix during 6 weeks. The probiotic strains were selected based on their anti-inflammatory properties in an earlier study [23]. Mice were randomized into six treatment groups with 10 mice in each as follows: 1. Veh-Ovx, 2. Veh-Sham, 3. L. Para-Ovx, 4. L. Para-Sham, 5. L. Mix-Ovx, 6. L. Mix-Sham (See also Figure 1). The L. strains were given in the drinking water at a concentration of 10^9^ colony-forming units (cfu)/ml while control mice received tap water with vehicle (glycerol). Water bottles were changed every afternoon. The survival of the L. strains in the water bottles was checked regularly and after 24 h the concentration dropped one log unit to approximately 10^8^ cfu/ml. Each mouse drank on average 4.5 ml water/day. After two weeks of probiotic treatment, the mice were either sham-operated or ovx under inhalation anesthesia with isoflurane (Forene; Abbot Scandinavia, Solna, Sweden). Four weeks after surgery, blood was collected from the axillary vein under anesthesia with Ketalar/Domitor vet, and the mice were subsequently killed by cervical dislocation. Tissues for RNA preparation were immediately removed and snap-frozen in liquid nitrogen for later analysis. Bones were excised and fixed in 4% paraformaldehyde.

### Ethics Statement

All animal experiments had been approved by the local Ethical Committees for Animal Research at the University of Gothenburg.

### Peripheral Quantitative Computed Tomography (pQCT)

Computed tomographic scans were performed with the pQCT XCT RESEARCH M (version 4.5B, Norland, Fort Atkinson, WI, USA) operating at a resolution of 70 μm, as described previously [24]. Cortical bone parameters were analyzed *ex vivo* in the mid-diaphyseal region of the femur.

### High-resolution µCT

**Figure 2:**
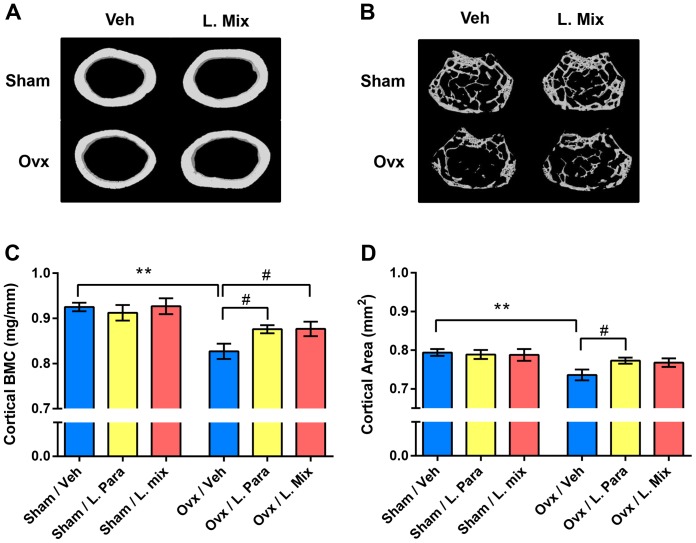
Probiotics Protect Mice from Ovx Induced Cortical Bone-loss. Eight-week-old mice were treated with either vehicle (veh), a single *Lactobacillus* (L) strain (L. para) or a mixture of three strains (L. mix) during 6 weeks, starting two weeks before ovx or sham surgery to study the preventive effect of probiotic treatment on ovx induced bone-loss. At the end of the experiment, dissected femurs were analysed with high-resolution µCT and peripheral quantitative computed tomography (pQCT). Representative μCT images of one cortical section from the veh and L. mix treated sham and ovx groups (A). Representative images of the trabecular bone volume (cortical bone excluded) from the distal metaphyseal region of femur (B). Cortical bone mineral content (BMC) (C) and cortical area (D) were measured by pQCT in the mid-diaphyseal region of femur. Values are given as mean±SEM, (n = 9–10). ** p≤0.01, * p≤0.05. Students *t* test ovx vs. sham. # p≤0.05, ANOVA followed by Dunnett's *post hoc* test within the groups, ovx L. Para and L. mix vs. ovx veh.

High-resolution µCT analyses were performed on the distal femur by using a 1172 model µCT (Bruker micro-CT, Aartselaar, Belgium). The femurs were imaged with an X-ray tube voltage of 50 kV and current of 201 µA, with a 0.5-mm aluminium filter. The scanning angular rotation was 180° and the angular increment 0.70°. The voxel size was 4.48 µm isotropically. The NRecon (version 1.6.9) was employed to perform the reconstruction following the scans. In the femur, the trabecular bone proximal to the distal growth plate was selected for analyses within a conforming volume of interest (cortical bone excluded) commencing at a distance of 538.5 µm from the growth plate, and extending a further longitudinal distance of 134.5 µm in the proximal direction. To illustrate the effect of probiotics on cortical bone in ovx mice, cortical μCT images of the diaphyseal region of one representative femur from each group were produced and are shown in Figure 2A. These CT images were derived from scans in the diaphyseal region of femur starting at a distance of 3.59 mm from the distal growth plate and extending a further longitudinal distance of 134.5 µm in the proximal direction. For BMD analysis, the equipment was calibrated with ceramic standard samples.

### RNA Isolation and Real Time PCR

Total RNA was prepared from cortical bone (femur with the ends removed and bone marrow flushed out with PBS before freezing) and bone marrow using TriZol Reagent (Invitrogen, Lidingö, Sweden). The RNA was reverse transcribed into cDNA using High-Capacity cDNA Reverse Transcription Kit (#4368814, Applied Biosystems, Stockholm, Sweden). RT-PCR analyses were performed using the StepOnePlus Real-Time PCR system (Applied Biosystems). We used predesigned RT-PCR assays from Applied Biosystems (Sweden) for the analysis of IL-6 (Mm00446190_m1), IL-1β (Mm00434228_m1), TNFα (Mm00443258_m1), RANKL (Mm00441908_m1), OPG (Mm00435452_m1), Osterix (Mm04209856_m1), Col1α1 (Mm00801666_g1), osteocalcin (Mm01741771_g1) and TGFβ1 (Mm03024053_m1) mRNA levels. The mRNA abundance of each gene was calculated using the “standard curve method” (User Bulletin 2; PE Applied Biosystems) and adjusted for the expression of 18S (4308329) ribosomal RNA.

### Serum and Urine Analysis

Analyses were performed according to the manufacturer's instructions for serum and urine calcium (Ca) (QuantiChrom™Calcium Assay Kit (DICA-500), Bioassays systems, Hayward, CA, USA), serum and urine creatinine (Mouse Creatinine Kit, Crystal Chem, Downers Grove, IL, USA), serum 25-Hydroxy Vitamin D (EIA, Immunodiagnostic Systems, Herlev, Denmark). As a marker of bone resorption, serum levels of C-terminal telopeptides were assessed using an ELISA kit (Nordic Bioscience Diagnostics, Herlev, Denmark). Serum levels of osteocalcin, a marker of bone formation, were determined with a mouse osteocalcin immunoradiometric assay kit (Immutopics, San Clemente, CA).

### Flow Cytometry

Bone marrow cells were harvested by flushing 5 ml PBS through the bone cavity of one femur using a syringe. After centrifugation at 473 g for 5 min, cells were resuspended in Tris-buffered 0.83% NH_4_Cl solution (pH 7.29) for 5 min to lyse erythrocytes and then washed in PBS. For flow cytometry analyses, cells were extracellularly stained with BD Horizon v450-conjugated anti-CD4 (Becton Dickinson (BD), Franklin Lakes, NJ, USA) and allophycocyanin (APC) anti-CD25 (BD). By using anti-Mouse Foxp3 Staining Set (eBioscience, Vienna, Austria), Foxp3 was intracellularly stained with Phycoerythrin (PE)-conjugated anti-Foxp3, according to the manufacturer's instructions. Regulatory T cells were defined as CD4^+^CD25^+^Foxp3^+^ and results are expressed as frequency of lymphocyte parent gate. Samples were run on a BD FACS Canto II and data was further processed using Flow Jo 8.8.6 software (Three Star Inc, Ashland, USA).

### Statistical Analyses

We used GraphPad Prism for all statistical analysis. Results are presented as the means ± SEM. Between-group differences were calculated using unpaired t tests, ovx vs. veh. Comparisons between multiple groups were calculated using a one-way analysis of variance (ANOVA) followed by Dunnett's test to correct for multiple comparisons, within the sham and ovx groups respectively. A two-tailed p≤0.05 was considered significant.

## Results

### Probiotic Treatment Protects Mice from Ovx-induced Cortical Bone Loss and Increased Bone Resorption

To determine the preventive effect of probiotic treatment on ovx-induced bone-loss, eight-week-old mice were treated with vehicle (veh), a single *Lactobacillus* (L) strain (L. para) or a mixture of three strains (L. mix) during 6 weeks, starting two weeks before ovx or sham surgery (Figure 1A). Uterus weight can be used as an indicator of estrogen status and ovx resulted in an expected decrease in uterus weight that was similar for all treatments (Table 1). In addition, ovx increased body weight, fat mass and thymus weight in all treatment groups (Figure 1B, Table 1).

**Table 1:** Organ Weights.

	Sham			Ovx		
	Veh	L. Para	L. Mix	Veh	L. Para	L. Mix
Uterus weight (mg)	45.9±4.8	65.9±12.5	65.2±10.4	8.95±0.7**	11.0±3.2**	6.9±0.3**
Gonadal Fat (mg)	371±41	296±40	326±40	597±68*	630±28**	577±58**
Thymus weight (mg)	55.5±3.2	53.6±5.1	47.7±2.6	93.9±4.2**	90.0±5.1**	73.7±5.2**#

Eight-week-old mice were treated with either vehicle (veh), a single *Lactobacillus* (L) strain (L. para) or a mixture of three strains (L. mix) during 6 weeks, starting two weeks before ovx or sham surgery to study the preventive effect of probiotic treatment on ovx induced bone-loss. Mice were 14-weeks-old at the end of the study, when tissues were dissected and weighed. Results are given as mean±SEM, (n = 6–10). ** p≤0.01, * p≤0.05, Students *t* test ovx vs. sham. # p≤0.05, ANOVA followed by Dunnett's *post hoc* test within the groups, ovx L. Para and L. mix vs. ovx veh.

In the vehicle treated mice, ovx decreased the cortical bone mineral content (BMC) and cortical cross sectional bone area in the mid-diaphyseal region of femur (p≤0.01, Figure 2A, C, D). Importantly, ovx did not reduce cortical BMC or cortical cross sectional bone area in the L. para or the L. mix treated mice (Figure 2A, C, D). Cortical BMC was higher in both L. para and L. mix treated ovx mice compared to veh treated ovx mice (p≤0.05, Figure 2C). We analyzed C-terminal telopeptides to determine if the preventive effect of probiotics on cortical bone was mediated by changes in bone resorption. Ovx increased serum levels of C-terminal telopeptides in veh treated mice (+45±11%, p≤0.05 over sham) but not in L. para treated (20±9%, non-significant) or L. mix treated (23±9%, non-significant) mice (Table 2). Bone formation, as indicated by serum osteocalcin, was not significantly affected by probiotic treatment (Table 2). Trabecular bone parameters (BV/TV and trabecular BMD) in the distal metaphyseal region of femur were significantly reduced by ovx in all treatment groups (p≤0.05, Table 2, Figure 2B). These findings demonstrate that probiotic treatment protects mice from ovx-induced cortical bone loss and increased bone resorption.

**Table 2:** Trabecular and Cortical Bone Parameters, Serum Bone Markers and Regulatory T cells in Bone Marrow.

	Sham			Ovx		
	Veh	L. Para	L. Mix	Veh	L. Para	L. Mix
**Trabecular Bone**						
BV/TV (%)	16.2±0.7	16.8±0.8	17.4±0.8	13.2±0.7**	14.4±0.6*	13.8±0.5**
BMD (mg/cm^3^)	322±9	331±12	344±8	285±9*	302±7*	298±7**
Tb Th (μm)	45.3±0.7	46.0±0.7	47.9±1.0	43.2±0.8	42.7±0.8**	44.6±1.0*
Tb N (mm^−1^)	3.6±0.1	3.6±0.2	3.6±0.1	3.1±0.1*	3.4±0.1	3.1±0.1*
Tb Sp (μm)	124±1	124±1	124±1	126±1	124±1	127±1
**Cortical Bone**						
Crt Thk (μm)	181±2	180±3	186±3	168±2**	173±2	176±2#*
Tt Ar (mm^2^)	1.96±0.02	1.96±0.04	1.86±0.04	1.92±0.07	2.00±0.03	1.92±0.03
**Serum Bone Markers**						
C-terminal telopeptides (ng/ml)	12.6±2.2	17.6±1.6	18.4±1.7	18.2±1.4*	21.1±1.6	22.6±1.7
Osteocalcin (ng/ml)	90.9±10.4	97.1±6.7	105.6±6.1	159.9±11.8**	142.1±7.9**	136.9±6.5**
**Treg (%CD4+Foxp3+CD25+)**	0.117±0.023	0.109±0.017	0.090±0.017	0.054±0.004*	0.069±0.008	0.070±0.014

Eight-week-old mice were treated with either vehicle (veh), a single *Lactobacillus* (L) strain (L. para) or a mixture of three strains (L. mix) during 6 weeks, starting two weeks before ovx or sham surgery to study the preventive effect of probiotic treatment on ovx induced bone-loss. Mice were 14-weeks-old at the end of the study, when tissues were dissected and weighed. Trabecular bone parameters were analysed by high resolution μCT in the distal metaphyseal region of femur; Trabecular bone volume as a percentage of tissue volume (BV/TV); Trabecular bone mineral density (BMD); Trabecular thickness (Tb Th); Trabecular number (Tb N); Trabecular separation (Tb Sp). Cortical bone was measured by pQCT in the mid-diaphyseal region of femur; Cortical thickness (Crt Thk); Total cross-sectional area inside the periosteal envelope (Tt Ar). The resorption marker, C-terminal telopeptides and the formation marker, osteocalcin were measured in serum. Femur bone marrow cells were stained with antibodies recognizing CD4, Foxp3 and CD25. Values represent the percentage of Treg (CD4+ Foxp3+ CD25+) of gated lymphocytes. Results are given as mean±SEM, (n = 6–10). ** p≤0.01, * p≤0.05, Students *t* test ovx vs. sham. # p≤0.05, ANOVA followed by Dunnett's *post hoc* test within the groups, ovx L. Para and L. mix vs. ovx veh.

### Probiotic treatment reduces expression of inflammatory cytokines and the RANKL/OPG ratio in cortical bone

**Figure 3:**
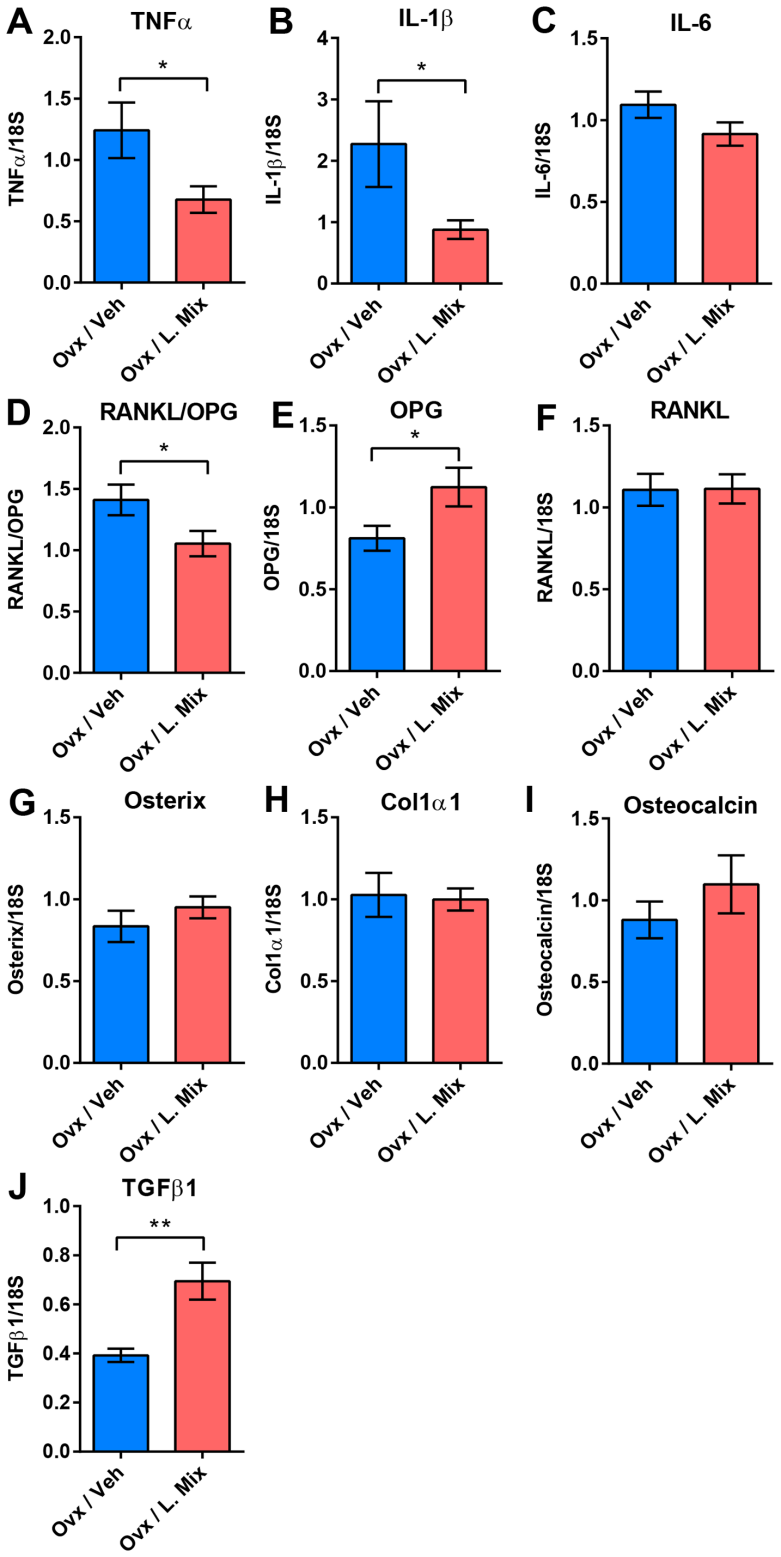
Probiotics Reduces Expression of Inflammatory Cytokines and the RANKL/OPG ratio in Cortical Bone and Increases Expression of TGFβ in Bone Marrow. QRT-PCR analysis of the expression of genes known to promote bone resorption; (A) Tumor Necrosis Factor alpha (TNFα), (B) Interleukin-1β (IL-1β), (C) Interleukin-6 (IL-6), (D) Ratio of Receptor activator of nuclear factor kappa-B ligand (RANKL) and Osteoprotegerin (OPG), and individual graphs for (E) OPG, (F) RANKL and genes known to promote bone formation; (G) Osterix, (H) Collagen, type I, α1 (Col1α1) and (I) osteocalcin in cortical bone and (J) transforming growth factor (TGF)β in bone marrow from 14-week-old ovariectomized (ovx) mice treated with either vehicle (veh) or a mixture of three probiotic *Lactobacillus* strains (L. mix) during 6 weeks, starting two weeks before ovx or sham surgery to study the preventive effect of probiotic treatment on ovx-induced bone-loss. Values are given as mean±SEM, n = 9–10. ** p≤0.01, * p≤0.05 versus veh treatment, Student's *t*-test.

To investigate the mechanism for the effect of probiotic treatment on ovx-induced cortical bone loss, we measured bone related mRNA transcripts in cortical bone (Figure 3). The mRNA levels of TNFα, an inflammatory cytokine produced by immune cells that promotes osteoclastogenesis, and IL-1β, a downstream regulator of the effects of TNFα on bone, were significantly decreased by probiotic treatment compared to vehicle treatment in ovx mice (TNFα −46%, p≤0.05; IL-1β −61%, p≤0.05, Figure 3A, B). The expression of IL-6 did not differ between treatments although there was a tendency to decreased expression in the probiotic treatment group (−20%, p = 0.12, Figure 3C).

The RANKL/osteoprotegerin (OPG) ratio is a major determinant of osteoclastogenesis and bone resorption. Importantly, probiotic treatment decreased the RANKL/OPG ratio (−45%, p≤0.05 compared with veh) and this was mainly caused by an increased OPG expression (Figure 3 D–F). In contrast, the mRNA levels of three osteoblast-associated genes, Osterix, Col1α1 and osteocalcin, were not significantly affected by probiotic treatment (Figure 3G–H).

### Regulatory T cells in Bone Marrow

Some of the anti-inflammatory effects exerted by probiotic bacteria are thought to be mediated via the induction of regulatory T (Treg) cells [25]. FACS analysis of bone marrow showed that the frequency of Treg (CD4^+^CD25^+^Foxp3^+^) cells was decreased by ovx in veh treated but not in probiotic treated mice (Table 1). Treg cells are dependent on TGFβ for their induction and maintenance and the expression of TGFβ1 in bone marrow was increased in bone marrow in probiotic compared to veh treated ovx mice (+77±19%, p≤0.01, Figure 3J).

### Mineral Metabolism

**Figure 4:**
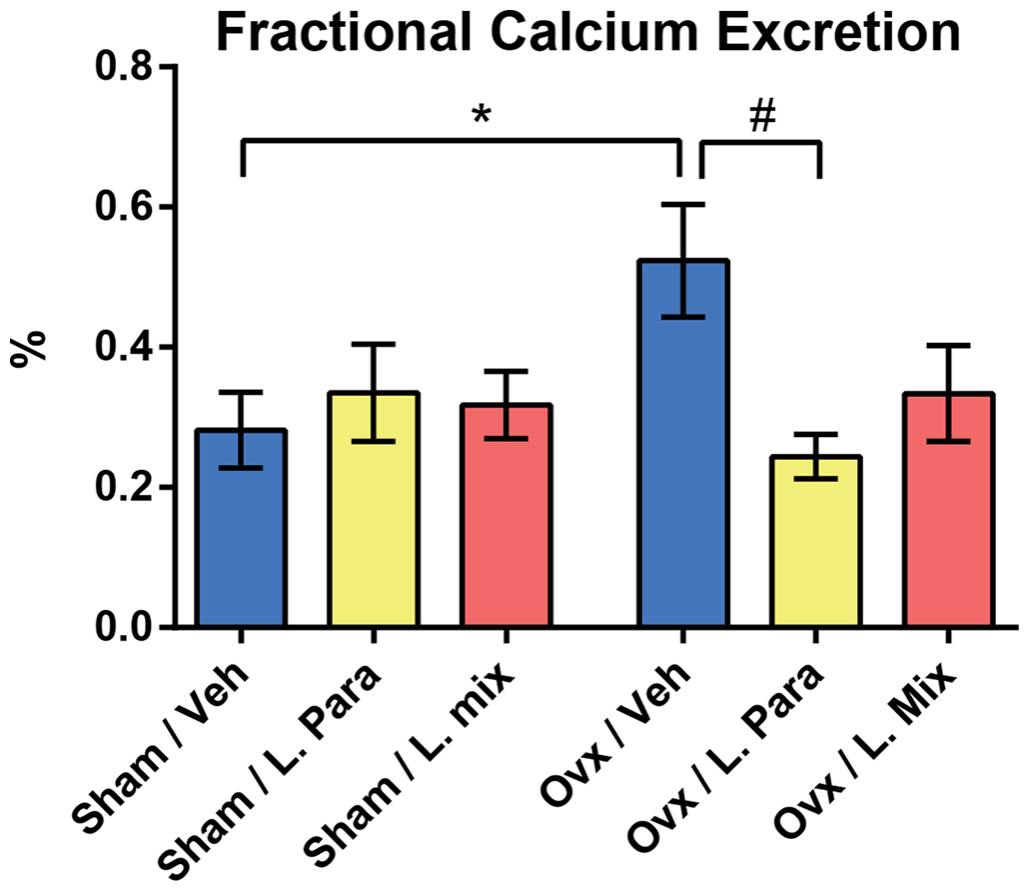
The Fractional Excretion of Ca was Increased by Ovx in the Veh Treated but not in the L. para or the L. mix Treated Mice. Ca and creatinine were measured in serum and urine from 14-week-old mice that had been treated with vehicle (veh), a single *Lactobacillus* (L) strain (L. para) or a mixture of three strains (L. mix) during 6 weeks, starting two weeks before ovx or sham surgery. Urinary fractional Ca excretion was calculated with the formula FECa  =  (urine Ca × serum creatinine)/(serum Ca × urine creatinine). Values are given as mean±SEM, n = 5–10 in each group. * p≤0.05. Students *t* test ovx vs. sham. # p≤0.05, ANOVA followed by Dunnett's *post hoc* test within the groups, ovx L. Para and L. mix vs. ovx veh.

The urinary fractional excretion of Ca (FECa  =  (urine Ca × serum creatinine)/(serumserum Ca × urine creatinine)) was increased by ovx in veh treated mice (+86%, p≤0.05, Figure 4). Interestingly, the ovx-induced increase in FECa was completely prevented by probiotic treatment (Figure 4). Serum levels of Ca were increased after ovx in veh but not probiotic treated mice (+13%, p≤0.05, Table 3). The urine Ca/creatinine ratio was not affected by ovx in any of the treatment groups (Table 3). 25-Hydroxy Vitamin D (25(OH)D_3_) in serum was not affected by ovx or probiotic treatment (Table 3).

**Table 3:** Mineral Metabolism.

	Sham			Ovx		
	Veh	L. Para	L. Mix	Veh	L. Para	L. Mix
Serum Ca (mg/dl)	9.1±0.4	9.2±0.4	8.5±0.3	10.3±0.4*	9.3±0.4	8.7±0.3#
Urine Ca/Creatinine Ratio	6.7±0.7	6.3±0.4	5.4±0.6	8.5±1.3	5.9±0.5	5.6±0.7
25(OH)D_3_ (ng/ml)	16.5±1.3	17.5±1.7	16.5±1.8	16.7±1.1	17.6±1.0	16.8±1.2

Calcium (Ca) and Creatinine were measured in serum and urine and 25-Hydroxy Vitamin D (25(OH)D_3_) was measured in serum from 14-week old mice that had been treated with vehicle (veh), a single *Lactobacillus* (L) strain (L. para) or a mixture of three strains (L. mix) during 6 weeks, starting two weeks before ovx or sham surgery. Results are given as mean±SEM, (n = 5–10). ** p≤0.01, * p≤0.05, Students *t* test ovx vs. sham, # p≤0.05, ANOVA followed by Dunnett's *post hoc* test within the groups, ovx L. Para and L. mix vs. ovx veh.

## Discussion

The GM regulates bone mass and probiotic treatment can affect the GM composition or the metabolic activity of the GM. In the present study we show that probiotic treatment protects mice from ovx-induced cortical bone loss. Both the L. para and the L. mix treatments protected mice from ovx-induced cortical bone loss and increased bone resorption. The urinary fractional excretion of Ca and the bone resorption marker C-terminal telopeptides in serum were increased by ovx in veh treated but not in probiotic treated mice, suggesting that the probiotic treatments reduced bone resorption in ovx mice. Probiotic treatment reduced the expression of the two inflammatory cytokines, TNFα and IL-1β, and increased the expression of OPG in cortical bone of ovx mice. These findings indicate that probiotic treatment alters the immune status in bone, resulting in attenuated bone resorption in ovx mice.

We have earlier shown that absence of GM leads to increased bone mass in GF mice and colonisation with a normal GM rapidly normalises bone mass [15]. The increased bone mass in GF mice was associated with an altered immune status reflected by decreased expression of inflammatory cytokines in bone. Estrogen deficiency increases inflammatory cytokines and reduces OPG in bone, resulting in bone loss [26]. Ovx mice have altered immune status and bone loss, resembling post-menopausal osteoporosis. Since probiotic treatment has the capacity to modulate the immune system, we hypothesized that probiotic treatment may attenuate ovx-induced increase in inflammatory cytokines and may, thereby, preserve the bone mass in ovx mice. The probiotic strains used, *L. paracasei* DSM13434 (L. para) or a mixture of three strains, *L. paracasei* DSM13434, *L. plantarum* DSM 15312 and DSM 15313 referred to as L. mix, in the present study were selected based on their anti-inflammatory properties in an earlier study [23]. In the study by Lavasani *et al* the selected lactobacilli strains had a suppressive effect on experimental autoimmune encephalomyelitis in an animal model of multiple sclerosis. L. para and L. mix prevented ovx-induced cortical bone loss to a similar extent. We can, therefore, conclude that treatment with L. para prevents ovx-induced cortical bone loss in mice while the possible independent roles of the two used *L. plantarum* strains for cortical bone remain to be determined. We believe that the main property of the L. para that explains the reversal effect of ovx on cortical bone is its anti-inflammatory capacity. However, further studies are required to in detail characterize its protective effect on ovx-induced cortical bone loss.

Cortical bone constitutes approximately 80% of the bone in the body and several studies demonstrate that cortical bone is the major determinant of bone strength and, thereby, fracture susceptibility [27]–[29]. Thus, the substantial cortical bone sparing effect of probiotic treatment in ovx mice in the present study indicates that this treatment might have the capacity to reduce non-vertebral fracture risk in postmenopausal women.

As expected, ovx increased bone resorption, as indicated by elevated serum levels of C-terminal telopeptides, in veh treated mice. In contrast, serum levels of C-terminal telopeptides were not significantly affected by ovx in probiotic treated mice. Bone formation, as indicated by serum osteocalcin was not influenced by probiotic treatment. Collectively, these findings indicate that the bone sparing effect of probiotics in ovx mice might be the result of attenuated bone resorption. A possible inhibitory effect on bone resorption is supported by the finding that the urinary fractional excretion of Ca was increased by ovx in the veh treated but not the probiotic treated mice. Estrogen therapy to post-menopausal women increases BMD associated with a reduced urinary fractional excretion of Ca [30], [31]. We propose that treatment with probiotics supresses bone resorption and as a consequence the urinary fractional excretion of Ca is decreased. However, we cannot exclude other effects of probiotics on urinary fractional excretion of Ca. In addition, it has been proposed that biochemical bone markers might be more influenced by trabecular than cortical bone. Thus, it is possible that effects on the release of biochemical bone markers from cortical bone to serum might be confounded by abundant release from trabecular bone. Therefore, further analyses of other more specific cortical bone parameters, such as osteoclast number in cortical bone, are required to confirm that the protective effect of probiotics on ovx-induced cortical bone loss is mediated via altered cortical bone resorption.

Mechanistic studies of the bone sparing effect of probiotic treatment in ovx mice revealed that the expression of several osteolytic cytokines such as TNFα and IL-1β as well as the RANKL/OPG ratio in cortical bone were suppressed by probiotic treatment. TNFα promotes osteoclastogenesis indirectly by stimulating RANKL expression by marrow stromal cells and osteoblasts and by direct stimulation of OCL precursors exposed to permissive levels of RANKL [32]–[34]. IL-1 is a downstream regulator of the effects of TNFα on osteoclastogenesis [35], [36]. The inhibitory effect of probiotics on the RANKL/OPG ratio in the present study was mainly due to an increased expression of OPG in cortical bone. OPG directly inhibits OCL differentiation at a late stage in a dose dependant manner [37]. Together, these findings indicate that probiotic treatment suppress osteoclastogenesis, resulting in reduced OCL-mediated bone resorption.

Treg cells are critical for maintaining self-tolerance and negatively regulate immune responses. In an earlier study, Lavasani et al demonstrated that the probiotic strains used in the present study induce Treg cells [23]. Several probiotic L. strains have been described to have a therapeutic effect in experimental mouse models of inflammatory bowel disease, atopic dermatitis, and rheumatoid arthritis associated with enrichment of Treg cells in the inflamed regions [25]. This inhibitory effect of probiotic L. strains was recently shown to depend on suppressive motifs in the DNA enriched in these strains that potently prevented dendritic cell activation and maintained Treg cell conversion during inflammation [38]. TGFβ is crucial for the induction and activity of Treg cells [39]. Interestingly, ovx decreased Treg cells in bone marrow in veh but not probiotic treated mice in the present study. Furthermore, the expression of TGFβ1 was increased by probiotic compared to veh treatment after ovx, suggesting that probiotic treatment prevents down regulation of Treg cells via an induction of TGFβ1. *In vitro* studies have shown that Treg cells directly inhibit OCL differentiation and function and that this effect of Treg cells is stimulated by estrogen and dependent on expression of TGFβ1 [40]–[42]. In addition, adoptive transfer of Treg cells decreases the number of OCLs and limits bone loss in ovx mice [43]. Collectively these findings may suggest that the suppressive effect of probiotic treatment on inflammatory cytokines and bone resorption involves effects by Treg cells.

In conclusion, treatment with L. para or the L. mix prevents ovx-induced cortical bone loss. Our findings indicate that these probiotic treatments alter the immune status in bone as demonstrated by reduced expression of inflammatory cytokines and increased expression of OPG, resulting in attenuated bone resorption in ovx mice. These data suggest a therapeutic potential for probiotics in the treatment of postmenopausal osteoporosis.
